# “I wanted to communicate my feelings freely”: a descriptive study of creative responses to enhance reflection in palliative medicine education

**DOI:** 10.1186/s12909-015-0465-4

**Published:** 2015-10-23

**Authors:** Lynn McBain, Sinéad Donnelly, Jo Hilder, Clare O’Leary, Eileen McKinlay

**Affiliations:** Department of Primary Health Care and General Practice, University of Otago, PO Box 7343, Wellington, 6242 New Zealand; Wellington Hospital, Riddiford St, Newtown, Wellington 6021 New Zealand; Mary Potter Hospice, 48 Mein St, Newtown, Wellington 6021 New Zealand

**Keywords:** Medical humanities, Creative work, Reflective writing, Family medicine, Medical student, Palliative medicine

## Abstract

**Background:**

The recent growth of arts and humanities in medical education shows recognition that these disciplines can facilitate a breadth of thinking and result in personal and professional growth. However creative work can be a challenge to incorporate into a busy curriculum. Offering the option of creative media as a way of reflecting is an example of how this can occur. This study aimed to examine the medical student response to being given this option to explore a visit to a patient in a hospice.

**Methods:**

This was a mainly qualitative study. In the 2012 academic programme, the class of 86 students were given the option of using a creative medium to explore their responses to both the visit and their developing communication skills. Students were required to write an accompanying commentary if submitting the creative work option. Sixty-four percent of the class chose a creative medium e.g. poetry, visual art, narrative prose, music. These students were asked to take part in research including completing a short on-line survey and consenting for their creative work and commentaries to be further examined. The creative works were categorised by genre and the commentaries analysed using inductive thematic analysis.

**Results:**

Seventeen students completed the on-line survey and fifteen consented to their work being used for this research. Thematic analysis of the student commentaries revealed the following themes: effectiveness for expressing emotion or ideas that are difficult to articulate; engaging and energising quality of the task; time for reflection; flexibility for individual learning styles and therapeutic value.

**Conclusions:**

Teaching the art of communicating at end-of-life is challenging especially when it involves patients, and teachers want to ensure students gain as much as possible from the experience. Offering the option to use creative media means that students can choose a medium for reflection that best suits them as individuals and that can enable them to benefit as much as possible from their experience.

## Background

In the last thirty years, the arts and the humanities have been introduced into the medical curriculum to broaden the education from its scientific focus and to develop the personal attributes of being a doctor [1]. This has taken many forms, ranging from using existing creative works as stimuli for discussion through to students creating their own work. Increasing numbers of medical schools feature arts-related activities, both in the U.S. [2] and in many other parts of the world [3–5].

Creative work has been introduced into health professional training for a variety of reasons. At its most basic, it can be used to reduce stress and increase positive emotions [6]. Creative work is also valued as a way to tap into tacit, pre-verbal ways of knowing [1, 7] and to enhance and promote reflection through the time required by the creative process [2, 3, 7]. Creative work can also be “a useful vehicle for taking a deeper look at ourselves and our practices, by engaging with metaphor and symbol” [7]. Producing creative work also has potential therapeutic value. It may enhance interactions with peers, increase enjoyment of study and thereby self-esteem, which results in more effective practitioners [3].

Areas of the medical curriculum in which students are asked or given the opportunity to produce creative work include internal medicine [8, 9] and primary care [5], and in modules involving visits to patients with chronic conditions [1, 10] or that focus on “Whole Person Care” [3, 7] or “Reflexivity in Professional Practice” [11]. We have not found any published work where such a programme is situated within palliative medicine education.

In most of the programmes described in the literature, a creative response is mandatory rather than an alternative to formal written work [1, 3, 7–9]. The goal of such programmes is often explicitly to foster the creative side of students as a way to enhance personal development. It is not clear whether the creative work in these programmes is formally assessed or is simply a course requirement. Only two programmes feature creative work as an option, with the alternative being an essay [5] or a commentary on a work of art [10]. Regardless of how creative work is used in medical training, it is acknowledged that there is a lack of research into its effectiveness or long-term impact [12–15].

Another key trend in medical education is a focus on reflection as a way to encourage students or practising health professionals to “explore their experiences in order to [gain] a new understanding and appreciation” [16]. Systematic reviews of the literature on reflection in the health professions have found little evidence or research into the most effective ways of facilitating high quality reflection [17, 18] although it has been argued that the ability to reflect can be developed over time [18].

In training for end-of-life care, it is acknowledged that students need time to “reflect and debrief about their own emotional reactions concerning the care of seriously ill and dying patients…[as] an essential element of the learning experience in palliative medicine” [19]. Students frequently approach this area of medicine with considerable concern and/or anxiety, particularly when influenced by previous personal loss [20, 21]. Reflection in this context can be usefully focussed on a real-time interaction between student and patient. However care is needed to ensure the reflection is authentic and meaningful rather than mechanistic where “we reduce this complex activity to a pragmatic and cyclical process, with subheadings and action plans that have limited use” [11]. The question then becomes what are effective ways to facilitate the necessary reflection especially when confronted with existential issues. The use of creative work has potential to meet this need [22].

Since it opened in 1979, Mary Potter Hospice has been involved in teaching palliative medicine to medical students in conjunction with the University of Otago Wellington [23]. The students are enrolled in a 6 year undergraduate medical degree programme which accepts a mix of new-entry undergraduates and those who have completed other degrees. The current palliative medicine module in year 4 consists of 1.5 hour tutorial sessions before and after a visit by student pairs to a patient in palliative care (at home or in a hospice). The assessed component has been a reflective essay which has been effective in encouraging students to integrate and articulate their personal response to the palliative care patient visit [20]. Recently, a change has been made to allow students to choose a creative medium instead of an essay. This was prompted by teaching staffs’ observations that the style of the writing produced by students was often creative rather than formal, as well as by an expressed desire by some students to use other creative media for their reflection. This synergy between teachers and students in 2012 led to the introduction of the option of a creative response, accompanied by a commentary of at least a page. The commentary was to include: reason for your choice of media; the relevance of the work to the assessment brief; how you have communicated your thoughts to the ‘audience’; any formal or informal sources that have influenced this work.

Sixty-four percent of the 86 students in 2012 chose the option to produce creative work instead of a formal reflective essay (55/86). The group comprised 28 males and 27 females and ranged from 21 to 47 years of age, which is demographically similar to the 2012 cohort.

A wide range of media were used as no limits were placed on this. The majority were in the form of poetry (25), visual art using paint, pencil, pastel or ink (13) or narrative prose (10). Smaller numbers chose music (3), photography (2), sculpture (1) and needlework (1).

This study sought to examine how the 2012 cohort group of fourth-year students responded to the option to use a creative medium to process their experience of visiting a patient at end-of-life and reflect on what they had learnt.

## Methods

Participants were recruited at the completion of the 2012 academic year from the cohort of 55 students who had chosen the creative option. Consenting students were asked to complete an anonymous on-line survey exploring their views on the value of the creative option and then at the survey conclusion were asked to consent to electronic copies of their creative work and commentaries being retained for research.

To minimise ethical concerns the research was timed to occur after the end of the academic year when final grades had been allocated; and measures were put in place to ensure neither the students nor patients were identifiable when the creative work was reported. The study was approved by the Otago University Human Ethics Committee (Health) (Ref. D12/310).

The analysis involved the survey responses being collated per question, the creative work categorised according to genre, and the commentaries analysed thematically. Analysis of the commentaries used a general inductive method where data are examined looking for responses held in common and those which are different [24, 25]. A two-stage data analysis process was undertaken. First, all authors independently reviewed the data, discerned categories and discussed possible themes. Second, one author (SD) developed the themes and presented these with example quotations to the author team and divergence in views was resolved by further discussion and testing of the data for other examples.

## Results

Seventeen survey responses were received from 55 students (31 %). Of the 17 students who completed the survey, 15 gave consent for their work to be used in research and publication. The demographics of the consenting group and genre of creative work is presented in Table 1.

**Table 1:** Demographics and genre of creative work

Entry into the undergraduate degree programme		Gender	
Undergraduate	6	Male	8
Graduate	9	Female	7
Ethnicity		Creative genre	
NZ European	7	Poetry	5
Maori	1	Narrative Writing	2
Middle Eastern	2	Pencil Drawing	2
British/Irish	1	Painting	1
British/Irish/Filipino	1	Sculpture	1
Italian/Other Asian	1	Needlework	1
Indian	1	Photography	2
Other Southeast Asian	1	Music	1

### The survey

All those who responded found it helpful having the option to use a creative medium and thought that a palliative care patient visit was an appropriate topic area for this. Almost all answered “yes” or “maybe” to the idea of an option to use a creative medium in other areas of their medical education.

### The commentary: emerging themes

The students frequently commented on the value of the creative option in their accompanying commentary. Analysis revealed several themes: effectiveness for expressing emotion, engaging and energising quality of the task, time for reflection, flexibility for individual learning styles, and therapeutic value.

### Effectiveness for expressing emotion or ideas that are difficult to articulate

Many students commented on how using a creative medium helped them to express emotions more effectively, especially those that are difficult to articulate or confront, or to express ideas that might otherwise be tacit. The latter is an acknowledged benefit of such an approach in adult learning [26].

I chose to write a poem for my palliative care assessment because it is a medium I have used in the past to express highly emotional experiences, particularly grief**.** (Student 12)

To a lot of people, music conveys emotions and feelings that words cannot express. By choosing music as my medium, I am acknowledging this concept. (Student 5)

Every once in a while, I undergo situations or encounter ideas where I find words unable to portray the overwhelming feeling associated with them. Being a very passionate photographer, I find that through photos I am able to convey at least part of the ideas that I want to express (Student 15).

**Fig. 1:**
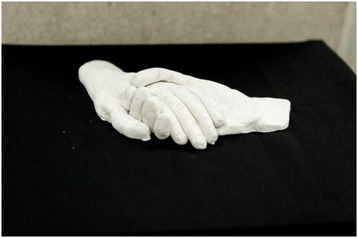
Clay sculpture (Student 9) “This work portrays the first contact made between a doctor and patient – the hand shake. I feel that this first physical contact helps to break down barriers, and assists in formation of the doctor-patient relationship. … The interlocked hands also symbolises the role of the doctor in patient management”

**Fig. 2:**
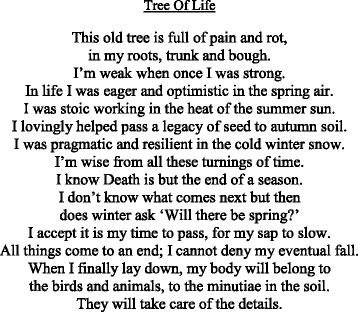
Poem (Student 4) “metaphorical references such as sunsets, winter or falling leaves suggest the transience of existence. Here I used the metaphors of a tree [’s] experience [,] seasons, slowing sap, falling and lying down. The pain and rot felt in the tree’s trunk and boughs represent Mr A’s pain in his spine and limbs from his cancer”

Some students particularly mentioned the value of metaphor to express ideas and feelings in their work:

I wanted to capture the idea that his ancestors were nearby and all around him, waiting for his return. This is symbolised by the figure standing behind him with a hand on his shoulder. This also portrays the support that strong spiritual beliefs can provide to patients at the end of life. (Student 13) Figs. 1 and 2.

A creative process can facilitate the making of connections and become metaphorical in itself, as shown in the following (note that fishing had been discussed with the patient visited):

I thought about the similarities between cross-stitching, fishing and how these can be applied to life itself. With cross-stitching, a simple pattern can take hours, or days, to complete. Each individual stitch by itself seems pretty meaningless, but together they all contribute towards the final result. (Student 11)

**Fig. 3:**
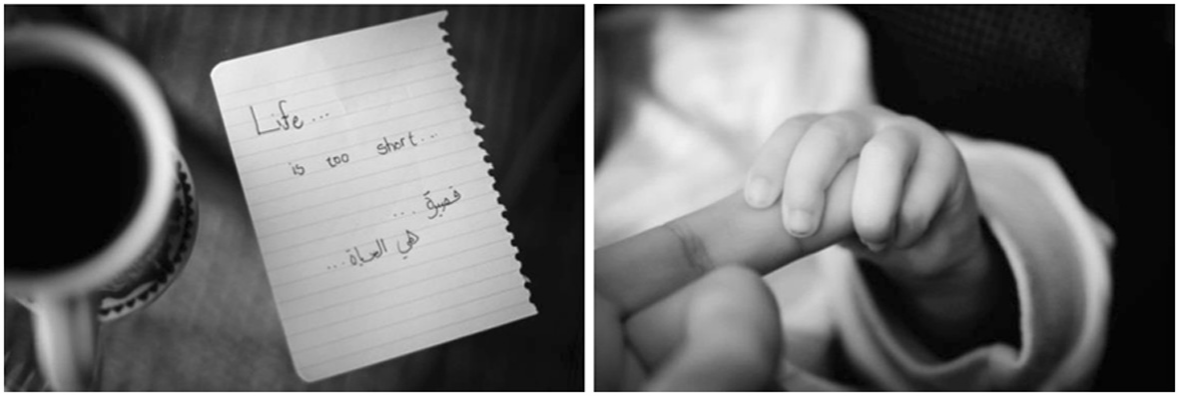
Photographs from a photo essay (Student 15): “A few days after taking some of the photos I was informed that my uncle had unfortunately passed away. Yes, I was deeply saddened by the incident. However, this stressed to me that indeed, life is too short”

Expressing emotions and ideas in a language that is not your native tongue is an added challenge for international students. The option to use creative media gave such students the freedom to bring in aspects of their heritage such as the Arabic script in Fig. 3, despite the fact that tutors may not necessarily be able to understand this. This emphasizes the primary importance of the task as something of value for the student, rather than merely a means of obtaining credit.

Another student felt comfortable including a traditional saying in Chinese script in his accompanying written work, together with an explanation.

### Engaging and energising quality of the task

**Fig. 4:**
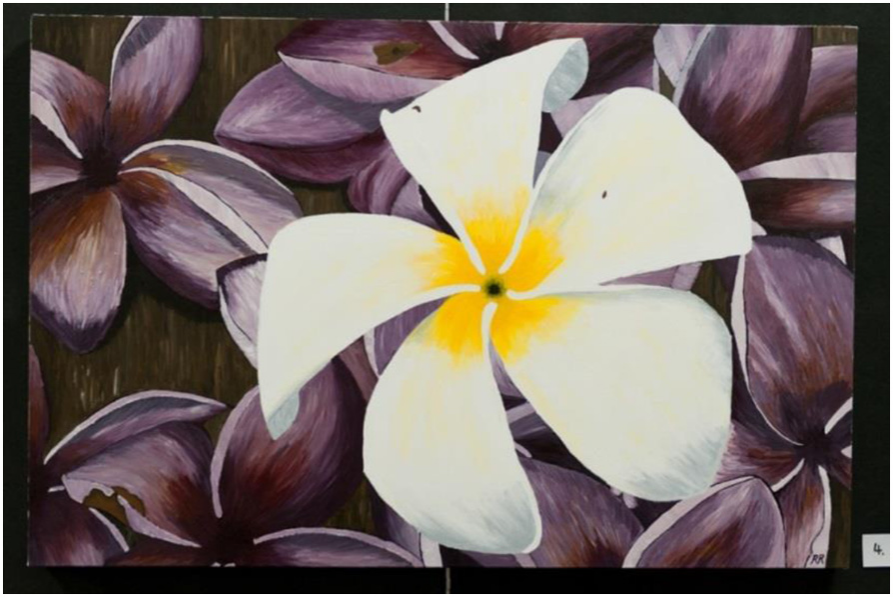
Painting of Frangipani flowers (Student 10): “I feel as though [they] have been snatched out of their primes, when they are only just reaching their full potential as professionals, parents and human beings. To me it is exactly like plucking the white flower in full bloom at the centre of the painting … more typical palliative care patients are represented by the background flowers…”

One benefit of this approach is that it may increase engagement and energise students [11]. Our students’ enjoyment of the task was sometimes palpable:

I loved to draw and paint as a child but any artistic promise was strongly discouraged for fear that I would want to make it my profession. When I heard … that we would not only be allowed but encouraged to do a creative piece for our assignment, I was understandably ecstatic. For me it has been a way to express myself in a form I used to cherish. (Student 10) Fig. 4.

For some, the sheer novelty changed the way they approached the task:

“If I was asked to write another reflective essay, it would be like a broken record in terms of assignments this year. But in doing something different (creative) and explaining in my accompanying document why I did it, made me reflect in a different way.” (anonymous survey response)

This latter comment also reflects how the option of a creative response can enhance the process of reflection.

### Time for reflection

Other students wrote of how the creative process enhanced their reflection purely through the way it forced them to slow down and spend time with their own thoughts while they worked:

This medium was chosen … as I felt the time spent in its creation would provide intimate time with my thoughts to reflect on my palliative care visit. (Student 9)

**Fig. 5:**
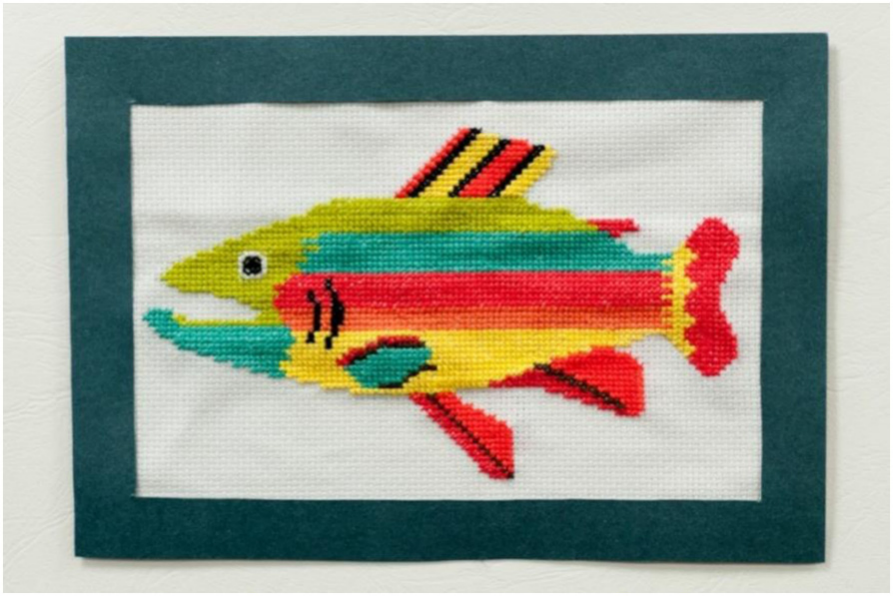
Cross-stitch of rainbow trout (Student 11) “I decided to use bright colours to reflect the happiness Mr X would have felt during his trout fishing escapades and also to reflect the power of our imaginations”

While I was doing the cross-stitch, I sat in the sun and listened to music. I felt truly relaxed. I think I will remember doing this assignment and how it made me feel. (Student 11) Fig. 5.

### Flexibility for individual learning styles

Seven student responses in the on-line survey indicated a dislike of writing essays as a reason to take up the creative option. Individual variation in preferred learning styles is a widely accepted concept and explicitly noted by one student as beneficial:

This visual type of work is best for helping me remember what I learnt. I am a visual learner who learns by looking at something. (Student 3)

For others, it can be the opportunity to explore a new medium for the first time:

As someone who considers them self to be non-creative and non-artistic, it took considerable time to think of an image that would represent communication between the doctor and patient. (Student 9)

The potential for individuality in the creative option means that students can choose something that is particularly meaningful to them; for example one student chose a medium that evoked memories of her grandmother.

For another student, the assignment proved to be a process of discovery in which the creative response he had planned turned out not to suit his purpose and he was then able to use the written reflection creatively to produce a piece which in itself lay outside of the typical reflective essay:

After putting the footage onto a video editing system and played around with it for a day or so I realised that the medium I needed to be working in for this project was not video but the written word. … I came to the conclusion that my real creativity was not the film I was trying to make but the reflection on it. (Student 6)

The flexibility of the assignment allowed students to find the type of activity that best fostered reflection for them as individuals. Even when one student found the creative response did not enhance their reflective process, another opportunity in the programme facilitated the goal of reflecting on and processing their experience to be achieved:

Unfortunately, the creative writing gave me limited opportunity to reflect upon the process of the interview.... Reflecting on the process of the interview was achieved in the reflective exercise that I completed after the hospice visit as well as in the actual writing of the assignment. (Student 2)

### Therapeutic value

Several students mentioned how the creative process had been therapeutic for them:

I found it a therapeutic process to explore these issues from the perspective of a patient who is dying. (Student 2)

It helped me conceptualize what was going on, and the image that it gave helped me cope both with her drawn out death and her eventual passing. (Student 12)

## Discussion

The use of a creative response in our palliative care programme has had benefits similar to those found in US and UK programmes, i.e. the enhancement of reflection [2, 3, 7], therapeutic value [3], and the facilitation of the expression of emotions or ideas that are difficult to express [1, 7]. The context in which we offered this creative opportunity–learning to talk to patients at end of life–is a particularly appropriate one since, in the words of one student, “palliative care and dying is a very emotion-filled-area”. This area of medicine is one in which it is important to process one’s own grief and to work out professional and personal boundaries. The faculty wanted students to comprehensively explore the visit to the patient, integrating their own personal responses, the patient’s story and the interactions that occurred between them. The students’ commentary that accompanied their creative work showed that, for those who chose this option, a creative response enabled them to achieve these goals in ways which may not have been accessible to them in writing a formal essay.

It is possible that creative responses can also be used to support medical students to develop critical thinking skills where information is interpreted and then weighed up to reach valid conclusions [27]. Similarly a creative response can be used to examine the application of ethical frameworks and concepts or to explore the application of science in medical education [28, 29].

The fact that a creative response was optional rather than mandatory sets our programme apart from many reported in the literature [1, 3, 8, 9]. We see the choice of media and flexibility of the assignment as a strength in that it gives students an opportunity to explore their experience in a way that best suits each individual. Allowing students the freedom to find their own way to make meaning helps to facilitate reflection [30].

Another reason for keeping the creative response optional is the context in which we are working. Although a mandatory creative exercise is important in some programmes [3], in a palliative care module, the task of meeting a dying patient is in itself challenging. We do not wish to doubly challenge students in both content and an unfamiliar medium for an assignment. The aim for the assignment is to encourage reflection and to support the students to process what they have experienced, not to explore creativity per se.

An elective approach also avoids some of the common challenges to the use of creative expression raised by Kumagai, such as resistance of students, and the need for additional curriculum time [1]. It also acknowledges and supports the fact that individual students have different learning styles, and this range of styles is in fact important within the field. We agree with Macnaughton [31] that “medical practice consists of a wide range of different jobs requiring many different sorts of people and the educational benefits of the humanities may not be appropriate for them all.”

One challenging aspect of this kind of assignment is to determine what sort of tutor feedback and assessment is best; remembering all students are required to complete this work with the goal of facilitating development of reflective practice. We use a combination of a group debriefing tutorial session and individual written feedback. This feedback is focused on the content of the commentary which describes how the creative work explores and conveys the student’s ideas and feelings and how it links with the creative work. The artistic quality of the work itself is not assessed.

In fact, assessment of the quality of personal reflection can be contentious in itself, particularly in relation to students’ emotional responses (regardless of the medium of expression) [17, 32]. Some students challenged this in their survey response, but this was unrelated to the use of a creative medium. We continue to informally evaluate whether our feedback to students is sufficient and appropriate and are moving towards making this assignment a course requirement that is not formally graded.

An unexpected positive outcome arising from the introduction of the creative option has been the successful staging of an exhibition of student work, “Te Toi Porehu: The Art of Palliative Medicine”, which gave an opportunity for the works to be more widely viewed by the various groups involved (hospice staff, the wider student body, faculty and relevant community members) [33].

### Limitations of the study

This mainly descriptive study reports on a sample from one student cohort in one location. No formal evaluation was conducted of the effectiveness of the method in comparison to the formal reflective essay alone, nor whether the mandatory nature of the patient visit and assessment impacts on student authenticity. All authors except JH teach in the medical student programme although not all are involved in palliative medicine education. The small sample also precludes any conclusions regarding potential differences according to age, gender or ethnicity, although those that chose the creative option were varied along these dimensions. This is something that could fruitfully be explored in future studies.

## Conclusion

The teaching of communication in palliative medicine is challenging and the opportunity to enable medical students to talk with patients at end-of-life although pragmatically difficult to arrange has proven to be an effective learning opportunity. Educationally, it is important to ensure students gain as much as they can in processing this experience. The introduction of an option to use creative media in a reflective response has been of value to the students in achieving this, particularly for those who are thus able to find a medium that suits their learning style. This approach would be suitable to use in other areas that have similar sensitive responses, such as maternity and fertility, aging, disability and mental health. We agree with Kumagai that there is a need for further *qualitative* research into the effectiveness of such approaches.
